# Extra-Genital Syphilitic Infection

**Published:** 1894-09

**Authors:** 


### EXTRA-GENITAL SYPHILITIC INFECTION.

THE following from the *Archiv. f. Dermat. u. Syph.* by Dr. R. Krefting appears in the *Am. Med. Surg. Bulletin :* The writer of this paper has taken his statistics from the University Clinic, of Christiania, Norway. During the twenty-five years ending December, 1891, 3,455 syphilitic patients were received, and 539 of them were cases of extra-genital infection. That is about $15\frac{3}{5}$ per cent. of all the cases. There were 61 men, 231 women, 247 children thus infected. There was a remarkable variation in the proportion between the genital and extra-genital cases in different years, running from 5 per cent. in 1891 to 34 per cent. in 1875. The variation is in part accounted for by the enforcing of police control of prostitution in the latter years, a larger proportion of genital lesions being sent to the hospital. Of the 539 cases, the location was noted in 280 cases as follows :

| | | | | | |
|---|---|---|---|---|---|
| Lips | were | affected | in ............. | 142 | cases. |
| Gums | " | " | " ............. | 1 | " |
| Tongue | " | " | " ............. | 11 | " |
| Throat | " | " | " ............. | 58 | " |
| Breast | " | " | " ............. | 58 | " |
| Chin | " | " | " ............. | 1 | " |
| Scalp | " | " | " ............. | 2 | " |
| Popliteal spaces | " | " | " ............. | 1 | " |
| Abdomen | " | " | " ............. | 1 | " |
| Finger | " | " | " ............. | 4 | " |

Of the lip lesions the upper lip was affected 76 times, the lower lip 49 times, the corner of the mouth 16 times, and both upper and lower lips once. They were always more or less crusted, and when the crust was removed there was left either an erosion or a deep wound of cartilaginous hardness, sharply cut outline, and brownish red color. The secretion is scant and thin; induration well marked and characteristic, excepting in children, when it is often wanting. The submaxillary and other glands in the neck on the side of the chancre are enlarged and of marked hardness.

Of the 58 throat initial lesions, 36 occurred on the tonsils, 15 on the right and 21 on the left. In one case both tonsils were affected. They gave rise to difficulty in swallowing, but not constantly. The swelling and hyperemia of the tonsil; the characteristic swelling of lymphatic glands of the neck; the ulceration of the tonsil often deep, gangrenous and covered with a grayish slough; the induration, sometimes wanting, gives the diagnosis. If both tonsils are affected, the diagnosis is much more difficult.

Of the 58 cases of breast affection, 19 times it was the right, 27 times the left, and 6 times both. The base of the nipple was the part most often affected, and it might be entirely surrounded by the lesion, though usually it extended only half way round. The lesions are usually covered with a bloody crust, which being removed exposes a dark red ulceration. Induration was always present; the axillary glands of the same side were always swollen and sometimes the glands along the pectoral muscle.

Of the 539 cases the manner of affection was ascertained in 439 cases, and it was found that in four-fifths of the cases it was by the mouth, either from eating and drinking utensils, kissing, nursing or smoking. Only five cases were traced to pipes or cigars. Three-fourths of the cases were by means of eating and drinking utensils. The course of the disease in most cases was less favorable than when rising from genital lesions.—*St. Louis Med. and Surg. Journal.*

---

Treatment of Chancre.—Dr. Willard Parker Worster claims (*Journal Cut. and Genito-Urin. Dis.*) that chancre is cured in the shortest possible time by the use of peroxide of hydrogen (Marchand) without pain or detention from business. The sore is sprayed with the peroxide under sixty pounds pressure and dressed with iodol powder, the same treatment being repeated. All the chancres reported were not by any means innocent, as some presented unmistakable signs of phagedena. The showing made by the author is certainly remarkable and worthy of trial.—O-D.—*St. Louis Med. and Surg. Journal.*

